# Fractal Correlation Properties of Heart Rate Variability as a Biomarker for Intensity Distribution and Training Prescription in Endurance Exercise: An Update

**DOI:** 10.3389/fphys.2022.879071

**Published:** 2022-05-09

**Authors:** Bruce Rogers, Thomas Gronwald

**Affiliations:** ^1^ College of Medicine, University of Central Florida, Orlando, FL, United States; ^2^ Institute of Interdisciplinary Exercise Science and Sports Medicine, MSH Medical School Hamburg, Hamburg, Germany

**Keywords:** heart rate variability, aerobic threshold, DFA a1, exercise intensity distribution, endurance training

## Abstract

While established methods for determining physiologic exercise thresholds and intensity distribution such as gas exchange or lactate testing are appropriate for the laboratory setting, they are not easily obtainable for most participants. Data over the past two years has indicated that the short-term scaling exponent alpha1 of Detrended Fluctuation Analysis (DFA a1), a heart rate variability (HRV) index representing the degree of fractal correlation properties of the cardiac beat sequence, shows promise as an alternative for exercise load assessment. Unlike conventional HRV indexes, it possesses a dynamic range throughout all intensity zones and does not require prior calibration with an incremental exercise test. A DFA a1 value of 0.75, reflecting values midway between well correlated fractal patterns and uncorrelated behavior, has been shown to be associated with the aerobic threshold in elite, recreational and cardiac disease populations and termed the heart rate variability threshold (HRVT). Further loss of fractal correlation properties indicative of random beat patterns, signifying an autonomic state of unsustainability (DFA a1 of 0.5), may be associated with that of the anaerobic threshold. There is minimal bias in DFA a1 induced by common artifact correction methods at levels below 3% and negligible change in HRVT even at levels of 6%. DFA a1 has also shown value for exercise load management in situations where standard intensity targets can be skewed such as eccentric cycling. Currently, several web sites and smartphone apps have been developed to track DFA a1 in retrospect or in real-time, making field assessment of physiologic exercise thresholds and internal load assessment practical. Although of value when viewed in isolation, DFA a1 tracking in combination with non-autonomic markers such as power/pace, open intriguing possibilities regarding athlete durability, identification of endurance exercise fatigue and optimization of daily training guidance.

## Introduction

In an attempt to determine physiologic thresholds for exercise intensity distribution, various methods have been employed such as gas exchange transitions, blood lactate and heart rate (HR) variability (HRV) kinetics (Meyer et al., 2005; Michael et al., 2017; Jamnick et al., 2020; Poole et al., 2021). The first boundary transition has been described as the aerobic threshold (AeT) represented by the first ventilatory (VT1) or blood lactate (LT1) thresholds (Meyer et al., 2005; Faude, 2009). The second boundary has been referred to as an anaerobic threshold (AnT) represented by the second ventilatory (VT2) or blood lactate (LT2) thresholds (Meyer et al., 2005; Keir et al., 2015). Threshold detection derived from HRV has been extensively investigated over the past 25 years since HR monitoring is a relatively straightforward, non-invasive, low cost option available to the general public (Michael et al., 2017). However, despite early enthusiasm, conventional HRV indexes have not been widely embraced for threshold or intensity assessment by either researchers, athletic monitoring device vendors, coaches or athletes. Part of this reluctance stems from two key issues. Traditional HRV indexes such as time domain parameters like standard deviation of corrected RR intervals (SDNN) or the standard deviation 1 from Poincaré plot analysis (SD1) do change during dynamic exercise but approach a nadir at around the AeT (Gronwald et al., 2020). Therefore, to determine the AeT, a formal incremental exercise test to near exhaustion is needed to demonstrate that nadir. Perhaps the more troublesome issue concerning a comprehensive intensity classification framework based on HRV is one of index dynamic range during endurance exercise. Since a nadir occurs at the AeT, there is no further information to be gained from observation past this region, making them poorly suited for spanning the full intensity range of exercise. Conversely, a dimensionless index of HRV based on fractal correlation properties (short-term scaling exponent alpha1 of Detrended Fluctuation Analysis: DFA a1) has been shown to have a wide dynamic range encompassing the low, moderate and high exercise intensity domains (Gronwald et al., 2020; Gronwald and Hoos 2020). Given these properties, a proposal was made to utilize this index as a biomarker for exercise intensity distribution including discrete numerical values that correspond to physiologic threshold boundaries (Gronwald et al., 2020). Since that hypothesis was made, some key findings have been published both supporting and expanding on this promising concept. This update will summarize progress to date and explore areas that still need further investigation.

## What are Fractal Correlation Properties of HRV?

Fractals are considered complex structures that possess self-similarity at various degrees of magnification. Natural spatial examples of fractal structures include coastlines, snowflakes or tree branchings. No matter the scale or magnification, the essential underlying pattern is similar (Eke et al., 2002). Fractal behavior of the cardiac beat series is characterized as degrees of self-similarity of the beat sequence over different time scales (Goldberger et al., 2002). DFA a1 is based on these fractal cardiac beat arrangements which can also be embodied as “correlation properties” of the pattern over short time spans. To better understand the concept of correlation properties, analogies to a random walk have been used (Hardstone et al., 2012). For example, during a random walk, at each next step, the walker can choose to go either right or left. If the choice the walker makes is not random but based on the previous sequence (series of right or left decisions), the pattern is described as being well “correlated” (DFA a1 near 1.0), since the future pattern is based on the past history. Values above 1.0 denote progressively higher degrees of correlation. But, if each new step is taken with equal, random chances of right or left, an “uncorrelated” pattern exists (DFA a1 of 0.5). During exercise it has been observed that at low intensity, DFA a1 values are usually in a correlated range near or above 1.0 (Gronwald et al., 2020; Gronwald and Hoos, 2020). As intensity rises, DFA a1 declines, passing 0.75 at moderate loads, continuing to drop further past the 0.5 range with increasing exercise intensity (uncorrelated random behavior of interbeat pattern), finally to drop below 0.5 (representing an anticorrelated range) at the very highest work rates. Anticorrelated behavior refers to a pattern that tends to bring the walker back to midline and can be viewed as an immediate self-correction mechanism associated with the potential failure of homeodynamic regulation and can only be tolerated for short time spans (Karasik et al., 2002). These correlation patterns are felt to be due to changes in sinoatrial pacemaker function under control by the balance between the reciprocal branches of the autonomic nervous system (ANS) (Michael et al., 2017). During exercise there is both a withdrawal of parasympathetic and enhancement of sympathetic activity resulting in a change of HRV including DFA a1 (White and Raven, 2014). Therefore, alterations in DFA a1 provide a view of autonomic balance from rest to severe intensity domains. This autonomic based index of systemic internal load contrasts with established markers of intensity that depend on physiologic subsystems such as cardiorespiratory variables (VO_2_, VCO_2_), chemical moieties (lactate) and measures of external load (speed/power).

## Do Specific Values of DFA a1 Correspond to Conventional Physiologic Exercise Thresholds?

Previous studies exploring DFA a1 behavior through progressive increases in exercise intensity have shown that at workloads near the AeT, index values fall midway between well correlated (1.0) and uncorrelated states (0.5) (Gronwald et al., 2020). Explanations for the association of physiologic breakpoints with correlation properties of HRV can revolve around practical observations (empirically derived from observing DFA a1 *vs*. VT1/LT1 during exercise ramp or stage studies) but can also be understood from a network physiology standpoint as an integrated concept of ANS regulation during endurance exercise (Balagué et al., 2020; Gronwald et al., 2020). Network physiology encompasses multiple neuromuscular, biochemical, peripheral and central nervous system inputs leading to an overall concept of “organismic demand” that is reflected in correlation properties of HRV and consequently in the response of DFA a1. Therefore, it was conjectured that a specific value of DFA a1 may correspond to the AeT, which would be helpful in sports and exercise science given the importance of this training boundary for intensity distribution models in many fields of application (Gronwald et al., 2020). Whether the desired program is polarized, pyramidal or threshold in type, identification of the low intensity boundary would be necessary (Seiler and Kjerland, 2006; Esteve-Lanao et al., 2007; Stöggl and Sperlich, 2015, 2019; Bourgois et al., 2019). With this objective in mind, the question of whether a value of DFA a1 between correlated and uncorrelated corresponds to the VT1 was evaluated in a group of male recreational runners (Rogers et al., 2021a). Results indicated that reaching a DFA a1 of 0.75 during an incremental treadmill test was associated with the VT1 and termed the heart rate variability threshold (HRVT). While this finding was encouraging, widespread application as an AeT boundary requires support in many demographic groups. Therefore, a very different class of participant, comprising male cardiac disease patients (congestive heart failure, stable coronary disease) was studied using an incremental cycling ramp protocol (Rogers et al., 2021f). During the cycling test the HR and VO_2_ attained at the VT1 was strongly associated with the HR and VO_2_ at the HRVT. Finally, in a contrasting population, the HR and cycling power at the HRVT was associated with the HR and cycling power derived from the LT1 in a group of elite triathletes (7 male, 2 female) performing an incremental cycling stage protocol (Rogers et al., 2022a). Although female data on DFA a1 behavior is sparse, the 2 female participants in this group had typical DFA a1 responses to incremental cycling exercise. In addition, it was also hypothesized that another physiologic breakpoint, the AnT, could occur at a DFA a1 of 0.5 (Rogers et al., 2021c). This value is associated with the transition from an uncorrelated to an anticorrelated pattern in HR time series. Since the anticorrelated state is felt to be an autonomic response indicating organismic destabilization (Seely and Macklem, 2004), it could correspond to a parallel phenomenon represented by a loss of cardiorespiratory sustainability. In support of this belief, study results from a recreational runner cohort showed that reaching a DFA a1 of 0.5 was associated with that of the VT2 and termed as the second heart rate variability threshold (HRVT2) (Rogers et al., 2021c). Most recently, a study done by Mateo-March et al. (2022) showed good agreement and correlation with both the HRVT and HRVT2 with lactate derived first and second thresholds in a large group of male professional cycling participants. Although the LT2 to HRVT2 correlation was high (r = 0.93 for cycling power, r = 0.71 for HR) there was a statistical difference in mean values (bias of 8 W or 4 bpm). As in Rogers et al. (2021c), the limits of agreement for the HRVT2 were relatively wide. Further studies should examine this more closely and attempts made to improve individual variations.

**FIGURE 1:**
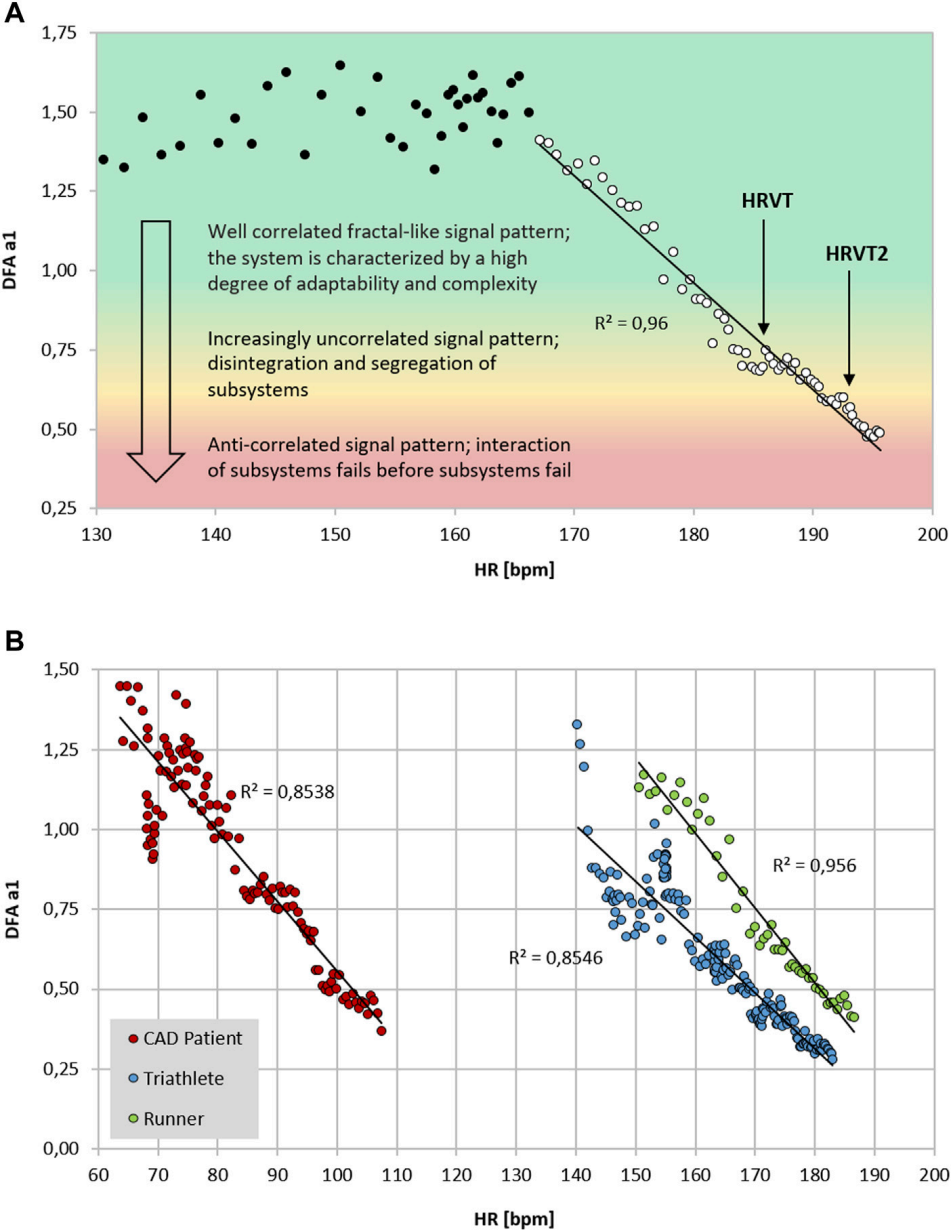
**(A)** DFA a1 *vs*. HR of a 26-year-old male runner with a VO_2MAX_ of 72 ml/kg/min, HR at VT1 of 183 bpm and HR of 192 bpm at VT2 performing an incremental treadmill ramp test, including a qualitative description of the signal pattern; data recorded with an ECG (MP36; Biopac Systems Ltd., Essen, Germany) (data from Rogers et al., 2021a; Rogers et al., 2021c). Data processed in Kubios HRV Premium software (Version 3.5) using automatic correction method (artifact percentage: < 5%). Shading indicates a 3 zone exercise intensity model defined by DFA a1 thresholds. **(B)** DFA a1 *vs*. HR of three participants during incremental exercise ramps (data from Rogers et al., 2021f; Rogers et al., 2022a; Rogers et al., 2021a respectively). Data processed in Kubios HRV Premium software (Version 3.5) using automatic correction method (artifact percentage: < 5%). Red circle: 59-year-old male with stable coronary artery disease (CAD), beta blocker usage and a VO_2MAX_ of 25 ml/kg/min, HR at VT1 of 83 bpm and HR at VT2 of 109 bpm performing an incremental cycling ramp test; data recorded with an ECG (VISTA Holter NOVACOR, Rueil, Malmaison, France). Blue circle: 23-year-old female triathlete with a VO_2MAX_ of 60 ml/kg/min, HR at LT1 of 154 bpm and HR at LT2 of 165 bpm performing an incremental cycling stage test; data recorded with Polar H10 chest strap (Polar Electro Oy, Kempele, Finland). Green circle: 19-year-old male runner with a VO_2MAX_ of 58 ml/kg/min, HR at VT1 of 167 bpm and HR at VT2 of 179 bpm performing an incremental treadmill ramp test; data recorded with an ECG (MP36; Biopac Systems Ltd., Essen, Germany).

Although prior studies indicated that DFA a1 declines as external exercise load rises, none had previously attempted to establish a distinct value corresponding to the AeT or AnT. Part of this difficulty relates to the method of DFA a1 plotting used during incremental testing. In previous work, DFA a1 behavior was routinely assessed by using non-overlapping measurement windows (of 1–5 min) often at the end of each intensity stage or condition of exercise (Gronwald and Hoos, 2020). Therefore, if an incremental exercise test consisted of 9 stages of 30 W per stage (10 w/min rise), only 9 DFA a1 data points would be available. To better detect a more precise pattern in DFA a1 behavior, a different method of DFA a1 plotting was utilized. This technique used fixed measuring windows of 2 min but did a rolling, ongoing recalculation every 5 s of activity (Kubios HRV Premium software “time varying” option: window width of 2 min, grid interval of 5 s). The 2-min time windowing was chosen based on the calculations by Chen et al. (2002) to achieve a sufficient number of RR data points to achieve DFA a1 validity. By using this method, a nearly straight-lined drop of DFA a1 from values of approximately 1.0 to 0.5 became apparent (Rogers et al., 2021a), providing an opportunity for simple linear interpolation of the corresponding HR or time plotted against DFA a1 of 0.75 (see Figure 1). Although not extensively studied, it also appears that constant power cycling intervals with 2-min measurement windows may also be used for HRVT determination (Gronwald et al., 2021). Another group looking at DFA a1 behavior in recreational runners using a 5-min measurement window showed similar correspondence to physiologic exercise threshold results during treadmill intervals with DFA a1 values of 0.68 ± 0.28 for the VT1 and 0.48 ± 0.11 for the VT2 (Naranjo-Orellana et al., 2021). Thus, DFA a1 values of 0.75 and 0.5 may represent a comprehensive solution to accepted physiologic exercise boundaries across a wide spectrum of individuals. In terms of individual participant agreement between HRV and gas exchange/blood lactate derived thresholds, they appear to be of similar magnitude to that of other comparisons of threshold approaches such as blood lactate versus ventilatory parameters (Pallarés et al., 2016), assessment of gas exchange techniques for VT1 determination (Gaskill et al., 2001), comparison of the maximal lactate steady state (MLSS) and functional threshold power (FTP) (Klitzke Borszcz et al., 2019) as well as the muscle oxygen desaturation breakpoint association to the MLSS (Bellotti et al., 2013). Despite the validation with established threshold concepts, it should be kept in mind that the present systemic approach is based on ANS regulation that does not necessarily match perfectly with other concepts based on subsystem parameters.

## DFA a1 Internal Load Assessment Beyond Physiologic Exercise Threshold Testing

Although exercise threshold awareness is of great importance, status of ANS regulation over time may provide key information about the level of internal load especially when the HR *vs*. VO_2_ or HR *vs*. power relationship is skewed. This is best exemplified by the process of eccentric cycling (Barreto et al., 2021). This activity is based on actively resisting motorized bicycle pedal motion moving in a reverse direction, thereby applying muscular force in an eccentric fashion. During eccentric cycling at the same VO_2_, an individual is usually able to pedal at 3x the power as conventional cycling. Therefore, at equivalent metabolic cost, eccentric cycling can be performed at much higher power levels, resulting in enhancement of muscular size, strength, and oxidative properties (LaStayo et al., 2000). This is particularly advantageous in groups with low cardiovascular fitness who would benefit from improved functional muscle mass such as those with heart failure or pulmonary disease (Gremeaux et al., 2010). Although there appears to be distinct benefits to eccentric training, assessing intensity distribution is problematic. This is due to both the power and HR discrepancy measured from conventional physiologic exercise thresholds compared to those determined by concentric cycle testing (Barreto et al., 2021). HR is usually higher at equivalent VO_2_ in eccentric compared to concentric cycling possibly associated with a reduction in cardiac stroke volume (Ritter et al., 2019). Additionally, many of the ideal candidates for eccentric training have cardiac disease and may be on beta adrenergic blocking medication, further complicating conventional HR targets. An initial exploration of DFA a1 behavior during prolonged low intensity unaccustomed eccentric cycling indicated that some participants had major suppression of DFA a1 into the anticorrelated range associated with substantial autonomic perturbation (Rogers et al., 2021d). Therefore, in exercise types that do not conform to standard exercise intensity models, DFA a1 could provide a measure of internal load and safety in “at risk” populations.

## Can DFA a1 be Used as an Index of Fatigue, Daily Training Guidance or Durability?

### DFA a1 as an Index of Endurance Exercise Fatigue and Daily Directed Training

Though recognized measures of endurance exercise induced fatigue have been explored, none are of practical value while in the midst of an exercise session or race. There are also questions regarding the validity of heart rate drift as a sign of fatigue during exercise (Maunder et al., 2021). HR drift appears to be a complex phenomenon (Souissi et al., 2021) that can normalize or even reverse with very long endurance efforts (Mattsson et al., 2011). Established performance indices or biomarkers such as counter movement jump (CMJ) height, running economy, muscle enzyme elevation like creatine phosphokinase (CPK), salivary hormones, markers of substrate availability, blood lactate concentration and cortical activity (Jastrzębski et al., 2015; Knechtle and Nikolaidis, 2018; Martínez-Navarro et al., 2019; Wu et al., 2019) are mainly used after the activity, during interval sessions or as monitoring tools at periodic times during standardized rest conditions. While regular HRV monitoring at rest has been proposed as a means to prevent functional overreaching and assess baseline autonomic balance (Stanley et al., 2013), no HRV index has been shown to have the ability to demonstrate fatigue while performing exercise. Since DFA a1 possesses dynamic range through all exercise intensity zones, a divergence between an indicator of autonomic status (e.g., DFA a1) and measures of external load (e.g., power/pace) could potentially be used as an indication of fatigue while still performing the activity. For instance, if the DFA a1 is usually 0.75 at a pace representing the AeT in a well-rested individual, would it be different after lengthy endurance exercise? To help answer this question an examination of running economy, HR, CMJ and DFA a1 in a group of experienced ultramarathon participants was explored before and after a 6-h trail based run (Rogers et al., 2021e). Seven athletes performed a 5-min treadmill test (at or below VT1 intensity) before and after the 6-h session. Results showed a significant DFA a1 decline from baseline after running for 6 h but without major change in HR or running economy. Additionally, mean DFA a1 in the post run group was 0.32, signifying an anticorrelated value seen with the highest intensity exercise domains despite a pace below the AeT. The fatigued state was confirmed by showing a significant impairment in CMJ height after the 6-h run. Interestingly, no HR change was noted in this study, similar to findings of Mattsson et al. (2011). Therefore, observation of inappropriately suppressed DFA a1 at an exercise intensity previously shown to be associated with well correlated values, can be potentially viewed as an autonomic indicator of fatigue.

In a monitoring purpose the usage of DFA a1 may help inform an athlete about their recovery from previous training sessions. It has become commonplace for individuals to monitor and track resting HRV as a method to direct daily exercise intensity and volume (Granero-Gallegos et al., 2020; Düking et al., 2021). Unfortunately, resting HRV requires a regular day-to-day monitoring routine including standardization (e.g., time of day, nutrition; Bellenger et al., 2016) and logistically may not fit into an irregular schedule. As resting HRV is a reflection of autonomic balance post exercise (Seiler et al., 2007), the observation of DFA a1 early in the upcoming exercise session could also provide similar clues. In other words, DFA a1 relative to power/pace could provide insight into current systemic recovery status while still in a low intensity warm-up stage. If relative suppression of DFA a1 is seen at a low exercise intensity previously associated with well correlated fractal patterns, it can be interpreted as signifying inappropriate autonomic balance. With this in mind, it is also important to realize that DFA a1 based physiologic exercise threshold testing performed in a fatigued, overreached or ill individual may not be similar to testing when healthy and well rested.

### Durability Assessment Using DFA a1 With Measures of External Exercise Load

**FIGURE 2:**
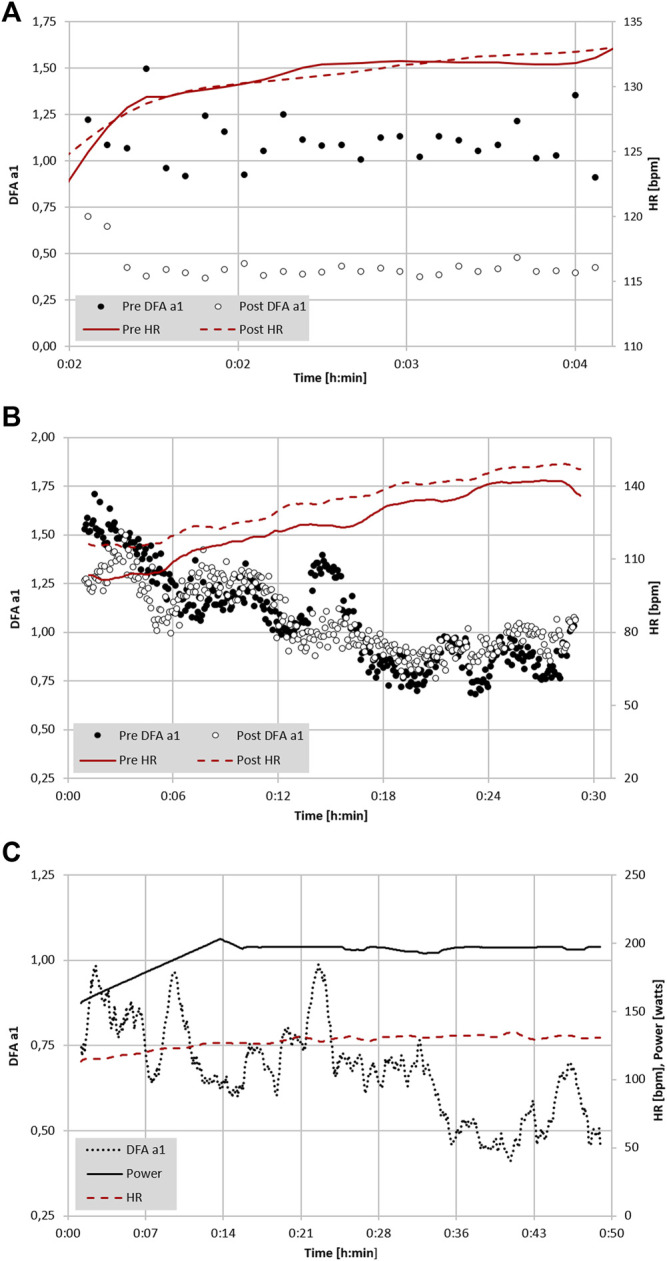
**(A)** Analysis of DFA a1 and HR of a 22-year-old male participant with a VO_2MAX_ of 74 ml/kg/min during a 5-min treadmill test at 65% of VT1 (VO_2_) before and immediately following a 6-h continuous trail run (data adapted from Rogers et al., 2021e). **(B)** Analysis of DFA a1, power and HR of a 41-year-old former Olympic male triathlete with a VO_2MAX_ of 65 ml/kg/min performing 6-min progressive cycling intervals of 100, 130, 160, 190, 220 W before and immediately after 2 h of continuous cycling exercise at 65% LT1 power (adapted from Gronwald et al., 2021). **(C)** Analysis of DFA a1 and HR of a 48-year-old male participant with a VO_2MAX_ of 46 ml/kg/min during 15 km cycling exercise at 90% of VT1 (VO_2_) (unpublished data). All data recorded with a Polar H10 chest belt (Polar Electro Oy, Kempele, Finland) and analyzed with Kubios HRV Premium software Version 3.5 (“time varying” option: window width of 2 min, grid interval of 5 s); lines represent 4 points moving average.

Extrapolation of the observation above could also lead to usage of DFA a1 as a marker of both “exercise durability” and as a method for daily decisions about “training readiness”. In a recent publication, athlete “durability” was described as “the time of onset and magnitude of deterioration in physiological-profiling characteristics over time during prolonged exercise” (Maunder et al., 2021). In other words, durability is an assessment of fatigue related reduction in performance, as opposed to standard measures of athletic fitness such as VO_2MAX_ or maximal lactate steady state. To assess durability, quantifying the starting point and degree of performance decline due to fatigue is needed. Leveraging DFA a1 as an index of overall “organismic demand” in conjunction with simultaneous measures of external exercise load could help assess this performance decline from an ANS perspective. In this context, DFA a1 would reflect changes/deterioration in autonomic balance seen at a particular pace/power during exercise. Since DFA a1 values span the spectrum of exercise intensity, the assessment of autonomic imbalance can be made whether the internal load condition is high or low. An example of this is shown in Figure 2A where DFA a1 is markedly suppressed at a pace well below the VT1 after the aforementioned 6-h run without changes in HR. In a second example (Figure 2B), there was no major change in DFA a1 behavior during incremental cycling exercise before and after a 2-h session performed at 65% of the LT1 power in a former Olympic triathlete (adapted from Gronwald et al., 2021). In the last example (Figure 2C), DFA a1 shows downward drift at an intensity just below the VT1 during a 15 km cycling session with stable HR in a recreational athlete. These three cases demonstrate the potential of DFA a1 to serve as an independent and systemic marker of fatigue and durability. The former Olympic triathlete showed no sign of DFA a1 “deterioration” after 2 h cycling at a low intensity, however the recreational athlete had declining DFA a1 at a stable power below the AeT, illustrating the potential interactions of inherent fitness level and external load over time.

In future studies looking at DFA a1 behavior at low to moderate intensities over longer time spans, incorporation of multipoint (rolling) averaging or longer measuring windows may lead to better comparative insights. Additionally, it will be important to examine the day-to-day variation and reproducibility of the DFA a1 versus external load relationship to determine what degree of precision can be expected for athletic assessments involving fatigue and durability.

## Limitations and Pitfalls

### Effects of Artifact Correction

Although the state of research related to DFA a1 and endurance exercise is encouraging, there are still potential limitations for this approach. Both the common occurrence of missed beat artifact and possible RR recording device bias could lead to erroneous DFA a1 values. Previous studies looking at missed beat artifact correction bias indicated variable effects on DFA a1 and none examined how HRV based exercise thresholds could be changed with increasing artifact presence (Stapelberg et al., 2018). In addition, the popular software Kubios HRV has two distinct varieties of artifact correction methodology (automatic and threshold artifact correction; Lipponen and Tarvainen, 2019), whose potential influence with respect to DFA a1 calculation or HRVT determination should be evaluated. To better understand these issues, we examined the effect of introducing progressive amounts of dropped signal artifact (by randomly deleting QRS complexes) to induce levels of 1, 3 and 6% artifact in otherwise ideal ECG tracings (Rogers et al., 2021b). Both Kubios automatic and medium threshold artifact correction methods were evaluated for bias. A negligible amount of bias was produced by 1 and 3% artifact correction with both methods. A larger amount of proportional bias (positive bias at low DFA a1, negative bias at high DFA a1) was seen with the threshold correction method at the 6% artifact level, rising to a maximum of 19% bias at very low, anticorrelated values of DFA a1. Fortunately, despite the varying degrees of bias seen, the HR at the HRVT did not differ between control (no artifacts) and any of the artifact groups or correction methods by more than 1 bpm. This is certainly reassuring regarding practical usage of HRV based exercise threshold determination and other fields of application.

### Recording Device Bias

Another aspect of the mentioned study about artefact correction (Rogers et al., 2021b) addressed whether the HRVT calculated from ECG was equivalent to the HRVT obtained by a chest belt (Polar H7) worn simultaneously. There was a small degree of bias at HRVT seen with the Polar H7, 4 bpm lower than that of the ECG. Several possibilities exist as to why there may be differences between devices. DFA a1 is a measure of RR related fractal correlation properties and therefore, specific patterns in the HR time series (Gronwald et al., 2020). Hence, a loss of RR resolution may lead to a failure to discern these patterns, leading to mistaken measures of fractal correlation properties. In support of this, a reduction in R peak detection precision has been shown to affect DFA a1 determination (Cassirame et al., 2019). According to Polar documentation, the R peak detection precision was enhanced in a next generation device, the Polar H10, implying that there was room for improvement in this property (Polar, 2019).

### ECG and Chest Belt Sensor Placement

**FIGURE 3:**
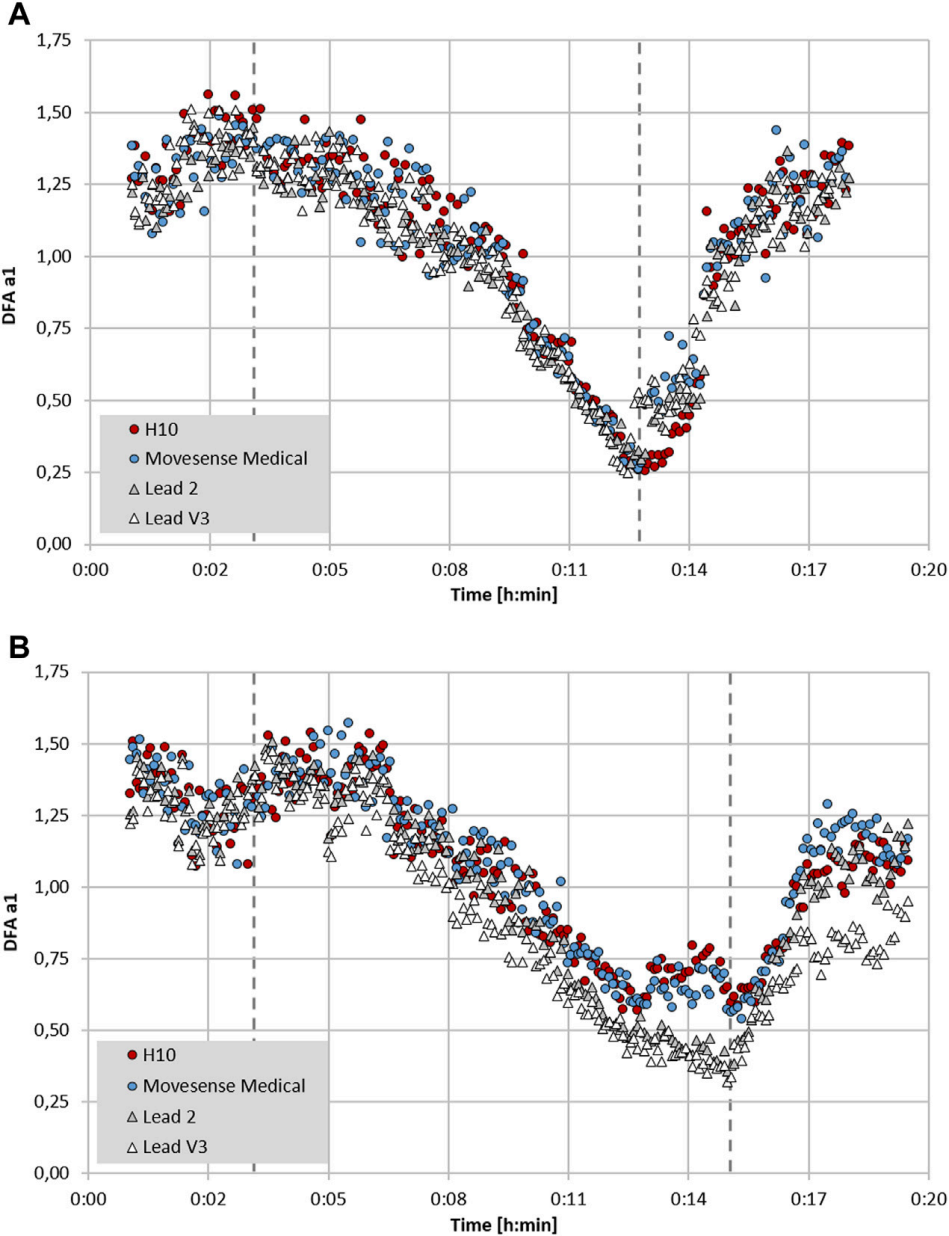
Plot of DFA a1 over time during the course of an incremental cycling ramp test until voluntary exhaustion, including 3 min of warm-up at 50 W and 5 min cool-down of unloaded pedalling (dashed lines mark the start of the incremental test and voluntary exhaustion), with two participants wearing a 12 channel ECG CardioPart 12 Blue (AMEDTEC Medizintechnik Aue GmbH, Aue, Germany; sampling rate: 500 Hz), Polar H10 chest belt (Polar Electro Oy, Kempele, Finland; sampling rate: 1,000 Hz) and Movesense Medical sensor single channel ECG chest belt (Movesense, Vantaa, Finland; sampling rate: 512 Hz). **(A)** Participant 1 with similar DFA a1 seen in both chest belt devices and lead 2 and V3 of the 12 channel ECG. **(B)** Participant 2 with discrepancy between DFA a1 in both chest belt devices compared to lead 2 and V3 of the 12 channel ECG. All data analyzed with Kubios HRV Premium software Version 3.5 (“time varying” option: window width of 2 min, grid interval of 5 s) (data and plot adapted from Rogers et al., 2022b).

In addition to a purely device based lack of precision, RR measurement may also depend on which particular ECG lead is chosen for analysis. In an intriguing study, significant variation in both RR measurement and conventional HRV indexes was seen depending on ECG lead selection (Jeyhani et al., 2019). After searching for standards regarding device validation studies for RR interval detection, there appears to be a lack of consensus on ECG lead selection as to what constitutes the “gold standard” (Task Force, 1996; Sassi et al., 2015; Dobbs et al., 2019). Chest belt recordings are most similar to ECG lead 2 (Jeyhani et al., 2019), but certainly are not identical. Although this has not been examined for DFA a1, an example may shed light on this issue (see Figure 3). Depending on the particular ECG lead analyzed, slightly different DFA a1 values but only minimal differences in HRVT determination can be seen (based on DFA a1 crossing 0.75). It is also possible that even with equivalent monitoring precision and R peak detection, changing sensor pad placement can alter DFA a1 measurement to a variable degree. This is apparent in Figure 3B where there is some divergence in data points between ECG lead 2 and V3 and both chest strap devices. This dissimilarity may stem from person to person variation in the cardiac axis, leading to slight ECG waveform changes depending on the signal sampling location (Jeyhani et al., 2019). Further study into this subject may help optimize DFA a1 measurement.

### Other Factors Affecting DFA a1 Behavior

From a network physiology standpoint, we must also keep in mind that DFA a1 is a reflection of net ANS activity and “organismic demand” of the systemic internal load. Therefore, changes in ambient temperature, fraction of inspired oxygen, intentional change of breathing frequency, hydration, nutritional status, and systemic illness can all play a role in modulating DFA a1 behavior. Lastly, though ANS balance appears to be the major determinant of DFA a1 behavior (Silva et al., 2015), locomotor-respiratory and cardiac coupling mechanisms and other non-neural mechanisms may also play a part (Persson, 1996; Qu et al., 2014). Finally, a major requisite of using pre-defined values of DFA a1 as a marker of exercise intensity is the need for uniform HRV software methodology in its calculation. One should also not expect exact similarity of results if different preprocessing methods from Kubios HRV are used for calculations (Voss et al., 2015).

## New Applications for DFA a1 Computation and Real-Time Display

While DFA a1 is an appealing physiologic biomarker, important factors such as accurate, low cost calculation and simplified data display are needed for widespread consumer usage. Over the past year, several web and smartphone applications have been developed to display DFA a1 independently of analysis software such as Kubios HRV. Websites such as “Runalyze.com” and “AIEndurance.com” utilize similar preprocessing (smoothness priors detrending), artifact correction (threshold approach) and time varying DFA a1 calculation methodology as Kubios HRV Premium for retrospective session analysis of DFA a1. Standard Garmin.fit files can be uploaded for analysis with estimation of HRVT based on linear regression using time varying computation of power/HR and DFA a1. The AIEndurance platform also tracks previous warm-up session DFA a1 to power relationships to estimate “readiness to train” as well as overall session deterioration in DFA a1 per unit power for measures of “durability”. Also of note is the availability of low cost smartphone apps that can process heart rate monitor data in real-time, providing a live view of DFA a1 status during exercise activities. A previous report described the use of the smartphone app “HRV Logger” (Android and iOS available) in both threshold determination and implementation of a polarized session program in a former Olympic triathlete (Gronwald et al., 2021). This particular application displays DFA a1 every 2 min sequentially without rolling window recomputation. A newer app called “Fatmaxxer” (Android available, https://github.com/IanPeake/FatMaxxer) enhances DFA a1 measurement capabilities even further. It includes features present in Kubios HRV Premium such as similar preprocessing (smoothness prior detrending), threshold correction and recalculation of DFA a1 e.g., every 5 s using 2-min rolling windows (time varying analysis: window width of 2 min, grid interval of 5 s). Lastly, “alphaHRV” a DFA a1 data field for Garmin devices (watches and cycling head units) is available in beta testing. It reports DFA a1 along with artifact percentage every second in real-time based on the prior 200 beats, bringing this metric to potentially millions of Garmin units. Having the ability to display accurate DFA a1 in real-time during an exercise session opens up a myriad of potential options, from on the spot threshold determination to assessment of fatigue status at the start (for daily directed training) as well as during a long exercise session (durability assessment and/or training interventions).

## Future Directions

A question that has eluded study thus far is whether DFA a1 behavior in male versus female participants is the same. Scant data exists at present showing HRVT similarity to the AeT in females as opposed to males. In addition, no information exists comparing DFA a1 response during exercise in pre- vs. post-menopausal women and along different phases of the menstrual cycle. From a theoretical standpoint, differences in DFA a1 response during exercise are possible in hormonally active women as several studies show altered HRV between the sexes (Abhishekh et al., 2013; Kappus et al., 2015). More specifically, autonomic balance appears to change over the course of the menstrual cycle (Brar et al., 2015) as opposed to a lack of change in cardiorespiratory parameters (Rael et al., 2021). An additional area of interest is HRVT validity in other sport-specific settings, especially regarding the influence of upper body activity on signal quality and differences in DFA a1 calculation (e.g., skiing, swimming, rowing), and over diverse age groups which would be helpful for widespread usage. In addition, consequences of intermittent positional bounce such as seen with running could be examined since they do have the potential to alter the cardiac axis, thereby affecting ECG waveform morphology (Astrom et al., 2003). Another question is whether the improvement of DFA a1 per unit of power or pace after a training intervention can indicate enhanced fitness, particularly in threshold boundaries. Preliminary data is suggestive (Rogers et at., 2021f), but additional research is needed. Prospective training intervention studies of participants who utilize knowledge of DFA a1 behavior during the early stage of routine exercise sessions for training guidance (as shown in monitoring with time domain HRV values during rest conditions (Düking et al., 2021)) would be of great interest as a tool to determine daily “readiness to train”.

## Conclusion

Studies of fractal correlation properties of heart rate variability during exercise have produced important findings over the past two years. DFA a1 behavior is a reflection of ANS regulation and is a component of network physiology which encompasses multiple neuromuscular, biochemical, peripheral and central nervous system inputs leading to an overall index of “organismic demand”. A distinct DFA a1 value of 0.75 has been shown to correspond to the aerobic threshold in recreational runners, elite triathletes and patients with cardiac disease. Further DFA a1 decline to the 0.5 range, signifying an uncorrelated random beat pattern, was associated with the anaerobic threshold, with both concepts representing limits of sustainable workloads. DFA a1 threshold determination can be affected by artifact correction and recording device bias but under most circumstances has enough resiliency to be of practical value with consumer grade heart rate monitors used for RR interval detection. Exercise modalities associated with difficulty in assessing intensity such as eccentric cycling may benefit from further investigation of DFA a1 as a marker of systemic internal load. Both web based and real-time smartphone tracking apps have been developed for DFA a1 monitoring and physiologic exercise threshold determination. Many of the current applications use similar computational methodology as the industry analysis standard of Kubios HRV software. Finally, while of value when even viewed in isolation, DFA a1 tracking in combination with external load markers such as power or pace open intriguing possibilities regarding athlete durability, identification of endurance exercise fatigue and optimization of daily training guidance.
